# The effect of progressive aerobic, resistance and stretching exercise combined with education on body weight among breast cancer survivors

**DOI:** 10.1080/07853890.2021.1896839

**Published:** 2021-09-28

**Authors:** A. Pinheiro, S. Silva, M. Sequeira

**Affiliations:** a Student of the 4^th^ year of Physiotherapy Degree of Superior Health School of the Polytechnic Institute of Setubal, Setubal, Portugal; b Teacher for the Physiotherapy Degree of Superior Health School of the Polytechnic Institute of Setubal, Setubal, Portugal

## Abstract

**Introduction:**

Breast cancer survivors have a high prevalence of excess body weight (65%) [1], which contributes to several comorbidities [2] and decrease in quality of life (QoL) [3]. The purpose of this study is to verify the effects of an exercise and education program on body weight, physical activity levels and quality of life of breast cancer survivors.

**Materials and methods:**

Thirteen breast cancer survivors took part of this case series study. They had a mean age of 69.8 (63–84) years, a body mass index (BMI) of 28.1 (24.4–32.3) kg/m^2^ and were lastly treated 7,6 (4-10) years ago. The program included a biweekly moderate-intensity aerobic, resistance and stretching exercise program, for 12 weeks, and educational sessions once a week for the first 4 weeks, regarding excess body weight and physical activity. The primary outcome was body weight and secondary outcomes were body fat, lean mass, knowledge of the topics discussed in the educational sessions, QoL (Global State Subscale of EORTC QLQ-C30), physical activity level (IPAQ), and program satisfaction. An informed consent was obtained from all participants.

**Results:**

Five of the eight eligible participants (62.5%) completed the study. All participants (5, 100%) decreased body weight, with a decrease in body fat and increase in lean mass. All of them also remained in the same physical activity levels, and all except one (4, 80%) improved QoL. Participants either maintained (1, 50%) or decreased (1, 50%) their knowledge on body weight, and either increased (2, 50%), maintained (1, 25%) or decreased (1, 25%) their knowledge on physical activity. All participants (5, 100%) were “highly satisfied” with the program.

**Discussion and conclusions:**

The authors conclude that 12 weeks of moderate-intensity aerobic, resistance and stretching exercise program improves loss of body weight, body composition and QoL, and helps maintaining physical activity levels, which is in accordance with previous literature. Effects on knowledge improvement were not observed, since there were only 2 educational sessions per topic, on a 4-week span, and the literature shows effects with longer programs. Furthermore, the collaborative methodology of the sessions led participants to interpret those as a moment to share experiences, instead of a moment to learn. These educational results may negatively influence the maintenance of the other results achieved. Similar programs should be provided, promoting these results to wider groups of breast cancer survivors, with educational sessions given more frequently and in a more informal setting, allied to exercise sessions.
